# *In-silico* analysis and expression profiling implicate diverse role of *EPSPS* family genes in regulating developmental and metabolic processes

**DOI:** 10.1186/1756-0500-7-58

**Published:** 2014-01-22

**Authors:** Bharti Garg, Neha Vaid, Narendra Tuteja

**Affiliations:** 1 International Centre Genetic Engineering and Biotechnology, Aruna Asaf Ali Marg, New Delhi 110 067, India

**Keywords:** EPSP synthase, Genome wide analysis, qPCR, Shikimate pathway

## Abstract

**Background:**

The EPSPS, EC 2.5.1.19 (5-enolpyruvylshikimate −3-phosphate synthase) is considered as one of the crucial enzyme in the shikimate pathway for the biosynthesis of essential aromatic amino acids and secondary metabolites in plants, fungi along with microorganisms. It is also proved as a specific target of broad spectrum herbicide glyphosate.

**Results:**

On the basis of structure analysis, this *EPSPS* gene family comprises the presence of EPSPS I domain, which is highly conserved among different plant species. Here, we followed an *in-silico* approach to identify and characterize the *EPSPS* genes from different plant species. On the basis of their phylogeny and sequence conservation, we divided them in to two groups. Moreover, the interacting partners and co-expression data of the gene revealed the importance of this gene family in maintaining cellular and metabolic functions in the cell. The present study also highlighted the highest accumulation of *EPSPS* transcript in mature leaves followed by young leaves, shoot and roots of tobacco. In order to gain the more knowledge about gene family, we searched for the previously reported motifs and studied its structural importance on the basis of homology modelling.

**Conclusions:**

The results presented here is a first detailed *in-silico* study to explore the role of *EPSPS* gene in forefront of different plant species. The results revealed a great deal for the diversification and conservation of *EPSPS* gene family across different plant species. Moreover, some of the EPSPS from different plant species may have a common evolutionary origin and may contain same conserved motifs with related and important molecular function. Most importantly, overall analysis of *EPSPS* gene elucidated its pivotal role in immense function within the plant, both in regulating plant growth as well its development throughout the life cycle of plant. Since EPSPS is a direct target of herbicide glyphosate, understanding its mechanism for regulating developmental and cellular processes in different plant species would be a great revolution for developing glyphosate resistant crops.

## Background

Aromatic amino acids and aromatic compounds are the essential components for the plant as well as for microorganism survival and hence their biosynthesis via shikimate pathway is crucial for their continued existence. EPSPS (5-enolpyruvylshikimate-3-phosphate synthase, EC 2.5.1.1.9), is considered as the sixth crucial enzyme of the shikimate pathway, catalyzes the formation of 5-enolpyruvylshikimate-3-phosphate (EPSP) from shikimate-3-phosphate (S3P) and phophoenolpyruvate (PEP) in the chloroplast [1] where EPSPS, a product of this pathway, acts as a precursor for the biosynthesis of aromatic amino acid in plants and microorganism [2,3]. Two types of EPSPS from different organisms have been classified [4,5]: type I EPSPS synthases, which are mainly found in all types of plants and bacteria and are naturally sensitive to herbicide named glyphosate (GLP; N-Posphonomethyl- glycine), and type II EPSPS synthases, that have been isolated from naturally occurring specific forms of microbes and are tolerant to glyphosate. The two types of EPSPS were found to share less than 30% homology with respect to their amino acid sequences. The identification of EPSPS as primary target of the broad spectrum non-selective herbicide glyphosate has generated immense interest in characterization of the enzyme [6]. Glyphosate can starve the plants of aromatic amino acids in most of the crops and weeds by competitively inhibiting the binding of EPSPS with phosphoenol pyruvate (PEP). Moreover, glyphosate also inhibits the import of cytoplasm synthesized EPSPS protein to chloroplast, which is the site of synthesis of aromatic amino acids. However, mere overexpression of *EPSPS* has been found to be incapable in confering glyphosate tolerance to the transgenic plants [7]. Therefore, altered EPSPS protein, with mutations in the key residues in the binding site could render EPSPS protein incapable of binding to glyphosate, have been identified. Recent researchers have exploited these altered *EPSPS* to design transgenic plants that have higher tolerance to herbicide, glyphosate, as compared to the wild type plants [8-11]. As a breakthrough study, overexpression of *Salmonella typhimurium EPSPS* mutant (Pro101to Ser) was reported to provide glyphosate tolerance in tobacco [12]. A mutant of rice *EPSPS* (Pro106 to Leu) conferred better glyphosate tolerance to *Escherichia coli* (*E. coli*) and tobacco transgenic plants [13]. Alteration of single amino acid residue (alanine 100, instead of highly conserved glycine found in other naturally occurring plants and bacteria) made CP4 EPSPS (from *Agrobacterium* sp. Strain CP4) insensitive to glyphosate [4]. Recent insights also proved that double mutations in type I *EPSPS* of *E. coli* and tobacco (threonine to isoleucine at position 97, proline to serine at position 101) leads to shift in glycine residue (at position 96) essential for glyphosate binding, eventually leading to glyphosate tolerance [4]. Substitution of proline residue to serine at position 106 of *Eleusine indica* (goosegrass) EPSPS protein has been predicted to provide five-fold higher capability for glyphosate resistance than wild type plants [14].

Structurally, the 3-D structure analysis of *E. coli* EPSPS synthases has revealed that the enzyme consists of six aligned parallel alpha-helices in each of two similar EPSPS I domains. Their pattern of alignment creates a specific electropositive attraction for anionic ligands at an interface between the two domains [15]. The nature of active sites, especially of the glyphosate binding cleft of EPSPS synthase has remained highly unresolved. Besides that, after comparing the crystal structures of *E. coli* EPSPS synthase during formation of either binary complex with S3P or formation of ternary complex with S3P and glyphosate elucidated that, the two domain containing *E. coli* EPSPS enzyme closes on ligand binding, thus, forming the active site in the inter-domain cleft. Glyphosate inhibition was considered as competitor with respect to PEP binding to occupy its site, though the molecular mechanism for such as specific inhibitory action of this inhibitor on EPSPS synthase is still obscure [16,17].

Although, some of the members of *EPSPS* gene family have been identified and characterized in model plants such as tobacco and *Arabidopsis thaliana* (hereafter termed as Arabidopsis), a systemic approach of comparative *in-silico* analysis among diverse group of species is still lacking. In the present study, we have identified and comprehensively analysed the *EPSPS* gene family across the diverse group of species. The work involves the identification of *EPSPS* gene family and analysis of their gene structure, conserved motifs and phylogenetic relationship. By taking the advantage of available expression data in genevestigator for *EPSPS* genes, we also performed a comprehensive analysis of tissue specific expression of *EPSPS* gene in plants, underlying its interesting role in plant development and under different stresses. Furthermore, time-course glyphosate treatment and subsequent quantitative PCR (qPCR) analysis unveiled the tissue specific expression pattern of *EPSPS* gene in tobacco. Ultimately, these findings will lead to potential applications for the improvement of glyphosate resistance in tobacco via genetic engineering.

## Result and discussion

Sequence retrieval by data base mining of *EPSPS* genes yielded 91 genes from different plant species. Further filtration by decrease redundancy software resulted in 58 non-redundant, unique sequences of *EPSPS* genes, which were further used to obtain their molecular weight and pI. (Table 1). Since extensive information is available for fully sequenced Arabidopsis and rice as the model species, therefore, these two were used in this study. The average molecular weights of EPSPS proteins from rice and Arabidopsis were 54.3 and 49.0 respectively, while, the pI values in rice and Arabidopsis *EPSPS* genes ranges from 5.00-9.88 and 5.98-9.28, respectively (Table 1). These results show high divergence between the EPSPS proteins even within the same plant species. Using SignalP, most of the EPSPS proteins from both rice and Arabidopsis were predicted to localize to chloroplast and cytosol with one rice EPSPS predicted to be present in mitochondria and the secretory pathway (Table 1). With the exception of this protein, all the other predictions support the hypothesized localization given by Dello-Cioppa et al. [18], however, experimental evidence of EPSPS protein localization is still pending to be explored in future.

**Table 1:** **Representing *EPSPS *** **coding genes in different organisms**

**Source**	**Accession**	**CDs**	**AA length**	**pI**	**MW (kDa)**	**Predicted localization**
*Lolium multiflorum*	DQ153168	1316	406	10.65	45.3	Chloroplast
*Lolium rigidum*	AF349754	1041	287	6.03	36.6	Cytosol
*Hordeum vulgare*	AK377052	462	153	6.72	16.8	Cytosol
*Triticum aestivum*	EU977181	1533	510	5.99	53.7	Chloroplast
*Triticum aestivum*	AK333537	1837	580	11.22	65.9	Chloroplast
*Bracypodium distachyon*	XM003557194	1533	510	7.56	55.8	Chloroplast
*Lolium rigidum*	AJ310166	957	299	8.91	33.3	Cytosol
*Sorghum hapalanese*	HQ436353	1335	444	5.30	47.3	Cytosol
*Oryza sativa*	NM001063247	1548	515	8.04	54.3	Chloroplast
*Oryza sativa*	AF413082	1536	511	8.04	53.9	Chloroplast
*Oryza sativa*	AB016765	1176	391	5.82	41.7	Cytosol
*Eleusine indica*	AY395700	1338	446	5.52	47.3	Cytosol
*Sorghum bicolor*	HQ436352	1470	490	7.59	51.5	Chloroplast
*Zoysia indica*	GU256772	1176	391	5.95	41.8	Cytosol
*Oryza sativa*	AK242404	1184	375	8.93	42.5	Cytosol
*Oryza sativa*	AY324880	995	317	9.88	34.9	Cytosol
*Brassica rapa*	AY512663	1545	514	6.99	55.1	Chloroplast
*Dicliptera chinensis*	AF371965	1551	516	7.54	54.9	Chloroplast
*Petunia hybrida*	M21084	1551	516	7.96	55.5	Chloroplast
*Conyza canadensis*	FR872821	1572	523	6.48	55.8	Chloroplast
*Camptotheca acuminata*	AY639815	1560	519	8.22	55.5	Chloroplast
*Capsicum annum*	JN160845	1551	516	7.54	55.3	Chloroplast
*Gossypium histurum*	FJ440839	1233	410	9.06	43.3	Cytosol
*Vitis vinifera*	FQ394893	1496	466	10.10	53.1	Secretory
*Calystegia hederacea*	EU526078	1563	521	7.58	55.4	Chloroplast
*Solanum lycopersicum*	M21071	1330	437	8.45	47.0	Cytosol
*Arabidopsis lyrata*	XM00280124	1563	520	8.45	55.7	cytosol
*Amaranthus tuberculatus*	FJ869880	1551	516	6.30	55.3	Chloroplast
*Orychophragmusviolaceus*	AF440389	1557	518	7.03	55.2	Chloroplast
*Conyza canadensis*	FR872820	1341	447	5.19	47.5	Cytosol
*Convolvulus arvensis*	EU698030	1560	519	6.04	55.2	Chloroplast
*Conyza bonariensis*	EF200072	1563	520	7.05	55.4	Chloroplast
*Oryza rufipogon*	CU861700	1338	430	10.38	49.5	Mitochondria
*Conyza sumatrensis*	AY834207	670	216	8.14	24.4	Cytosol
*A,qrqnthus rudis*	AY545657	1338	446	5.76	47.3	Cytosol
*Arabidopsis thaliana*	AY086717	1071	357	5.25	38.1	Cytosol
*Arabidopsis lyrata*	XM002894095	1566	461	5.98	49.0	Chloroplast
*Arabidopsis thaliana*	BT022026	1572	523	6.30	55.8	Chloroplast
*Acorus gramineus*	AY545656	1563	520	6.30	55.7	Chloroplast
*Populus trichocarpa*	XM002301243	930	310	6.28	33.2	Cytosol
*Ricinus communis*	XM002511646	1557	518	7.51	55.6	Chloroplast
*Allium macrostemon*	DQ462442	1557	518	8.26	55.5	Chloroplast
*Arabidopsis thaliana*	AT1G48860	1569	522	7.01	55.7	Chloroplast
*Arabidopsis thaliana*	BX816702	1470	489	5.71	52.4	Cytosol
*Phaseolus vulgaris*	DQ813667	1081	360	5.30	38.2	Cytosol
*Plantago lanceolata*	AY545665	1569	522	5.40	56.0	Chloroplast
*Lolium rigidum*	GU594896	795	265	6.94	27.9	Cytosol
*Phragmites australis*	JN580998	233	77	8.86	77.7	Mitochondria
*Sarracenia purpurea*	AY545663	295	96	6.92	107	Chloroplast
*Arabidopsis thaliana*	BX815732	795	265	8.70	27.7	Cytosol
*Oryza sativa*	AP002542	1815	565	9.16	64.0	Chloroplast
*Oryza sativa*	AB052962	936	311	5.00	34.3	Cytosol
*Helianthus salicifolius*	AY54566	1272	423	7.69	43.8	Secretory
*Lolium rigidum*	EU350204	792	264	6.00	27.5	Cytosol
*Helianthus salicifolius*	AY545662	189	63	9.85	65.2	Cytosol
*Vitis vinifera*	XM003633875	792	264	5.56	27.5	Cytosol
*Lolium rigidum*	EU350205	1593	530	8.00	56.4	Chloroplast
*Lolium rigidum*	EU350202	189	63	9.85	65.2	Cytosol

### Phylogenetic analysis

**Figure 1:**
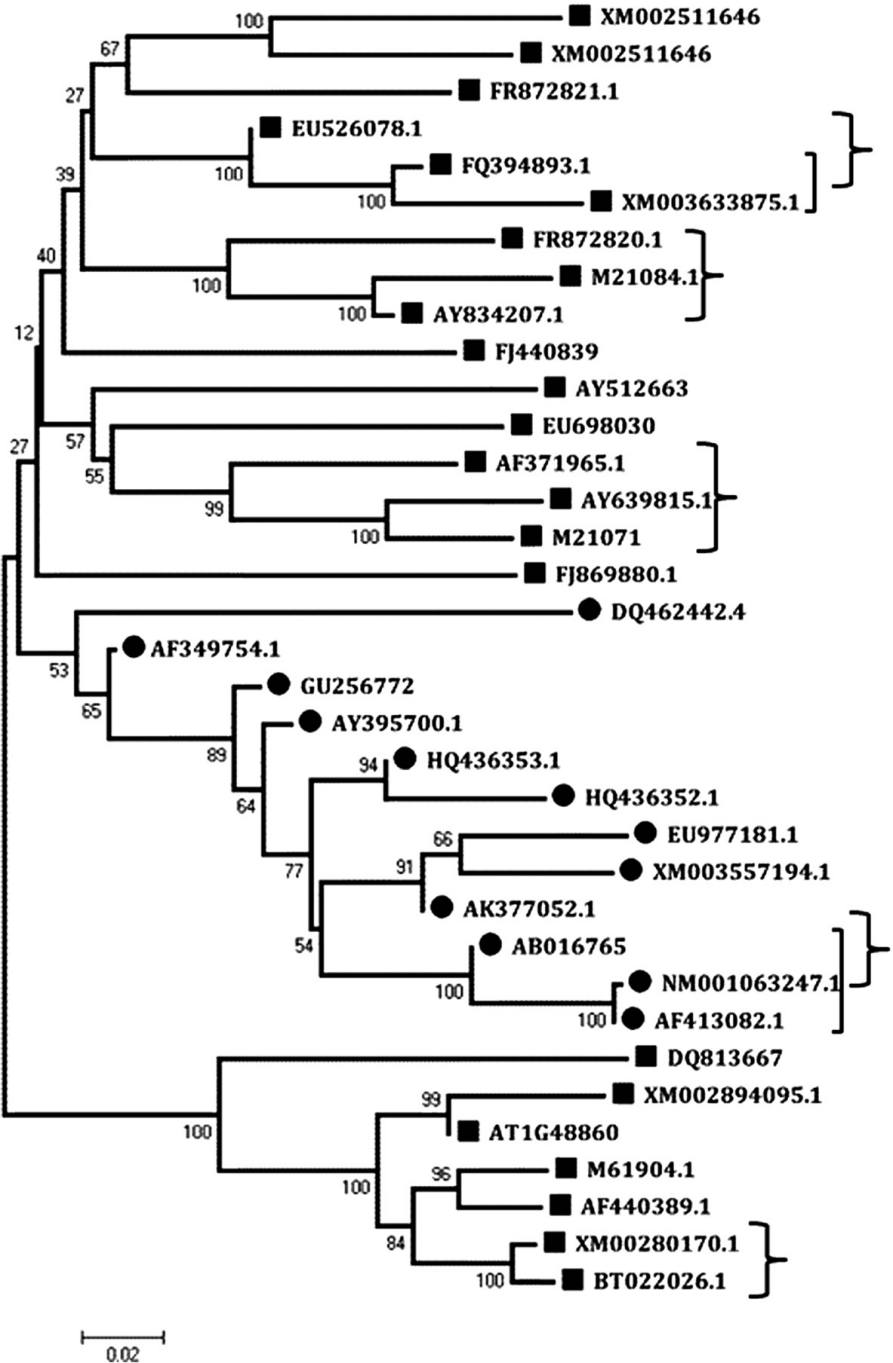
**Phylogenetic analysis of *EPSPS *genes from different plant species.** The phylogram was built from sequence alignment generated from NCBI COBALT and analyzed with MEGA5. The phylogram is bootstrapped with 1000 replicates. The circles represent the monocot plant species and the squares depict *EPSPS* genes from dicot species. The paralogue and orthologue gene pairs are marked with square and curly brackets respectively.

To analyze the phylogenetic relationship between *EPSPS* gene family members from various plant species, a phylogenetic tree, bootstrapped with 1000 replicates, was constructed using NCBI COBALT multiple sequence alignment tool. The phylogram divided the EPSPS proteins into two groups of monocot and dicot EPSPS, (Figure 1, represented by circles and squares, respectively). Although supported by low bootstrap value, this division could indicate towards divergent evolution of the *EPSPS* genes in monocots and dicots which probably implies that the proteins are interconnected in monocots and dicots with essential function that confers advantages to both of them. However the structural and functional importance of this divergent EPSPS evolution still remains unclear. The *EPSPS* phylogram supported with high bootstrap values, helped in identification of several paralogous (Figure 1, marked in squared brackets) and orthologous genes (Figure 1, marked in curly brackets).

### Analysis of conserved motifs

**Figure 2:**
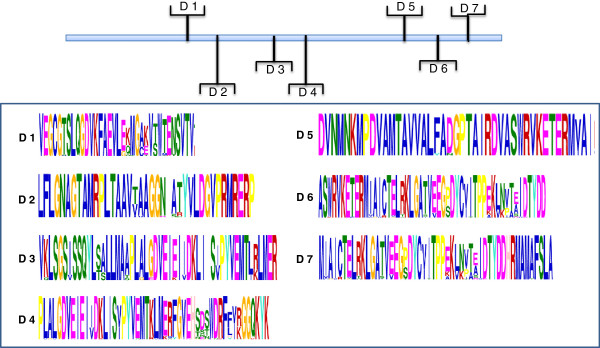
**Conserved domain analysis of EPSPS proteins.** The upper panel of the figure depicts the location of the domains while lower panel denotes the conserved sequences of the respective domains.

**Figure 3:**
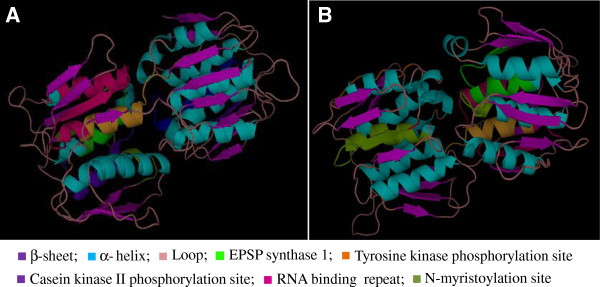
**Structural analyses of rice and Arabidopsis EPSPS proteins.** The 3-D structure analysis of **(A)** Rice and **(B)** Arabidopsis EPSPS proteins. The conserved domains of EPSPS protein have been marked in the figure.

**Figure 4:**
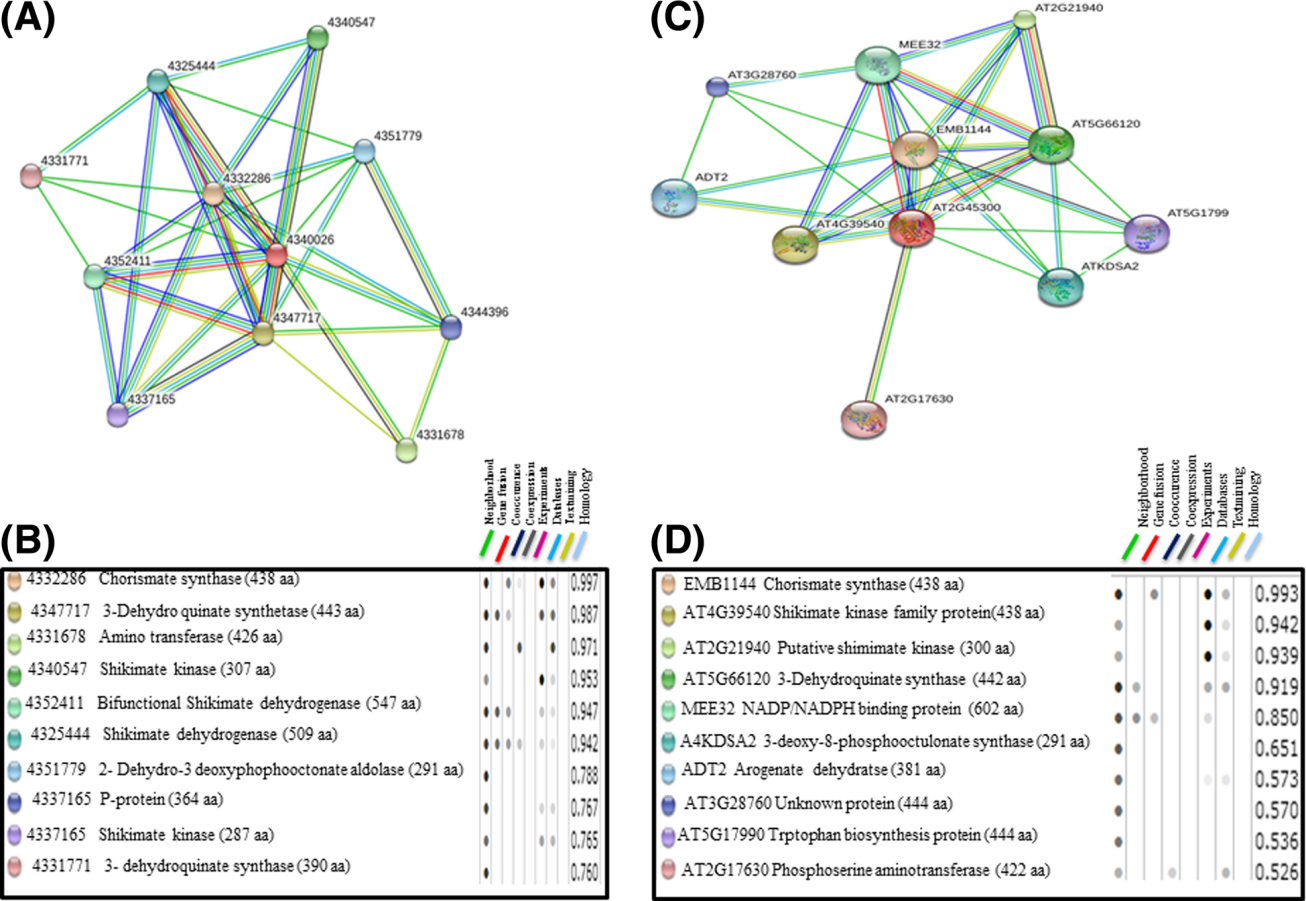
***In-silico *prediction of interacting partners for *EPSPS *gene by STRING. (A)** Figure showing Interacting partners for rice *EPSPS* gene. **(B)** The key to the putative interacting partners for rice *EPSPS* gene is listed. **(C)** Interacting partners for Arabidopsis *EPSPS* gene. **(D)**The key to the putative interacting partners of Arabidopsis ***EPSPS*** gene is listed. NM_001063247 and AT2G45300 were used as the input sequences to search for the rice and Arabidopsis interactions, respectively.

Amino acid alignment of *EPSPS* encoding genes from various organisms showed highly conserved regions (Supporting data, Additional file 1). The MEME suite GLAM2 version 4.8.0 was used to analyze the conserved motifs in the EPSPS proteins. A number of highly conserved motifs were observed in the EPSPS proteins from different plant species (Figure 2), indicating towards a strong conservation of these proteins during the evolution. These motifs could further provide deeper understanding that could help in gaining insights on the evolutionary relationships of plant *EPSPS* family. LP(G/S) KSLSNRILLLAAL and LFLGNAGTAMRPL motifs were present in almost all EPSPS plant species. These conserved residues of amino acids may function as the catalytic domains of EPSPS enzymes and are supposed to contribute in the glyphosate binding site. Similar motifs have been reported in bacterial EPSPS as well [14]. It has been proven that mutation of a single amino acid, especially lysine and arginine residue, can alter the binding site of glyphosate [19]. Besides that, substitution of an alanine residue for the second glycine residue in the conserved motifs could produce a mutant EPSPS, that exhibits a very low affinity for glyphosate [20]. To further visualize the conserved motifs of EPSPS proteins, 3-D models of rice and Arabidopsis EPSPS were generated using ESyPred 3D (web server 1.0) and visualized using PyMol. Figure 3 depicts different domains in rice and Arabidopsis EPSPS proteins as marked on their 3-D images. While, a common EPSPS I domain was found in both rice and Arabidopsis EPSPS proteins, EPSPS domain II was additionally observed in rice EPSPS protein sequence. As an exception rice harbours both of the EPSPS domains which probably indicate toward similar mode of action as in microbes. Furthermore, structurally, the EPSPS protein is composed of 35% α-helices, 17% extended sheets and 8% beta turn in rice, while Arabidopsis protein is composed of 31% α-helices, 19% extended sheets and 5% beta turn. This shows that the α-helices and the beta sheets cover comparatively larger portions of the 2-D and the 3-D structure in rice and Arabidopsis. The 3-D structure presented in the current study showed similarity with the previously observed studies wherein, bacterial EPSPSs have shown to fold in two globular domains and an inside-out α-β barrel domain with PEP-S3P binding in the interdomain cleft region [7]. In addition to that, the EPSPS interacting partners as well as its co-expression genes were also predicted in rice (Figure 4A and B) and Arabidopsis (Figure 4C and D) using String software. The analysis showed presence of several common proteins, such as chorismate synthase 2, 3-dehydroquinate synthase and shikimate kinase are found to be common interacting partners of EPSPS in both rice and Arabidopsis. Chorismate synthase catalyzes the last seventh step of the shikimate pathway which is conserved among the prokaryotes, fungi and plants for the biosynthesis of aromatic amino acids. Shikimate kinase is an ATP dependent enzyme, which catalyzes the phosphorylation of shikimate to shikimate 3-phosphate, it catalyzes the fifth step of shikimate pathway, 3-dehydroquinate synthase involves in the second step of shikimate pathway, which converts the 3-deoxy-arabinoheplutosonate 7-phosphate to 3-dehydroquinate, which have been shown to be essential for basic cellular metabolism machinery [21].

### Gene expression analysis

**Figure 5:**
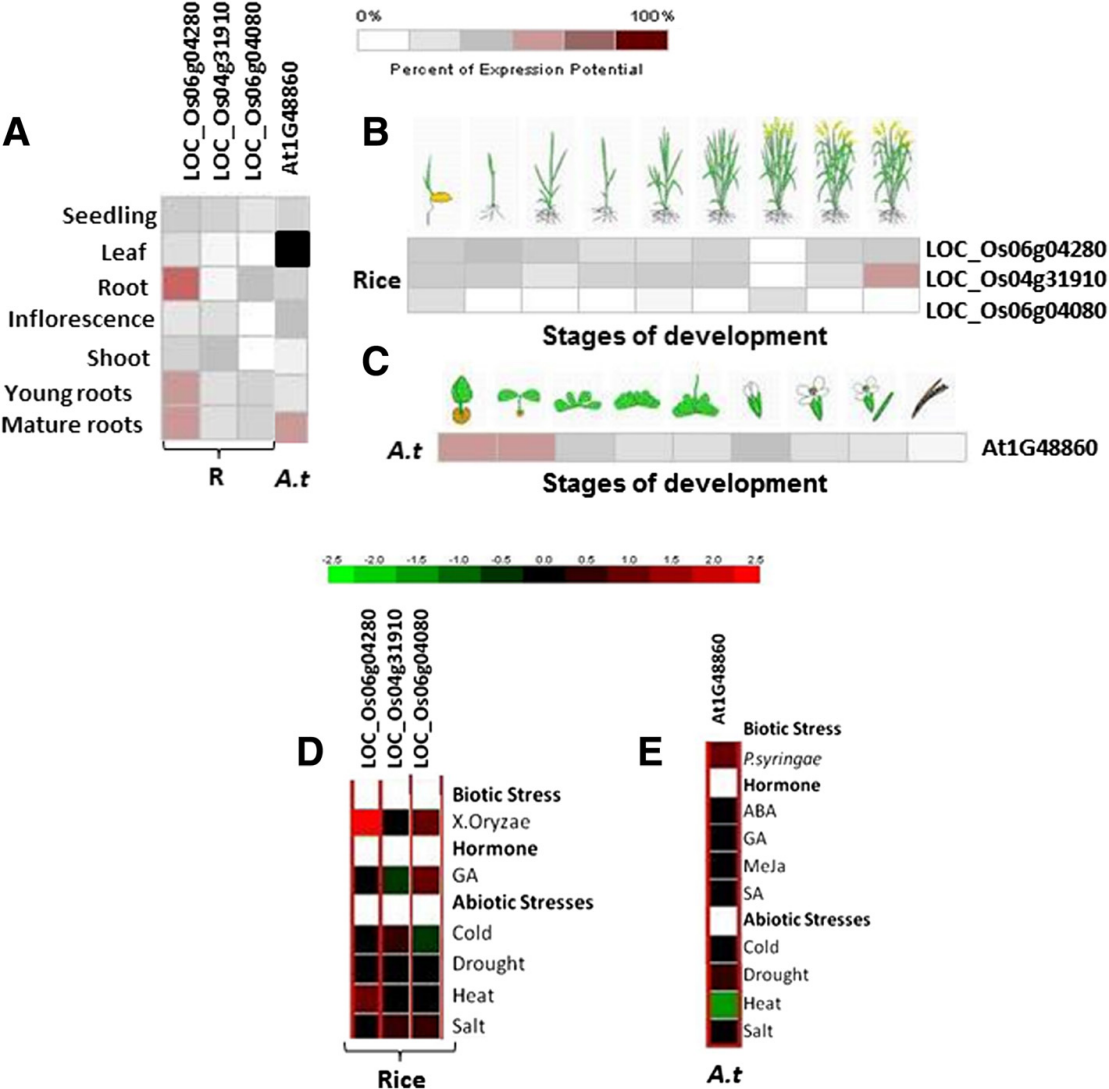
**Differential expression patterns of rice and Arabidopsis EPSPS proteins.** Heat map showing differential expression profile of rice (R) and Arabidopsis (*Arabidopsis thaliana*) *EPSPS* gene in different anatomical features **(A)**, developmental stages **(B & C)** and stress conditions and hormonal treatments **(D & E)**. Blackened block depicts absence of information on expression profile.

**Figure 6:**
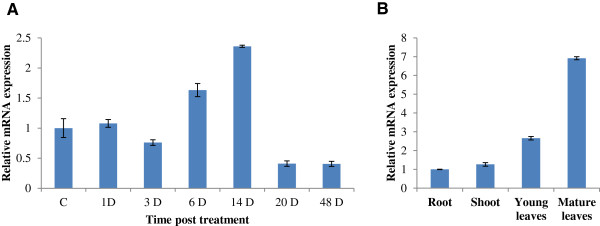
**Real time PCR analysis of EPSPS gene against glyphosate stress.** qPCR analysis of *EPSPS* transcript showing their regulation in response to glyphosate treatment **(A).** Real time PCR analysis of *EPSPS* during post treatment of 2.16 mg/l glyphosate at different time points. **(B).** Relative mRNA expression of *EPSPS* gene in different parts of plant. The relative mRNA expression for each transcript was calculated in comparison to control plants. Bars indicate the standard error (± SE) calculated from three independent experiments.

*In-silico* analysis of *EPSPS* gene expression profile in rice and Arabidopsis was performed using Genevestigator response viewer (https://www.genevestigator.com/). The data could be retrieved for three rice (LOC_Os06g04080, LOC_Os06g04280, LOC_Os04g31910) and one Arabidopsis (AT1G48860) *EPSPS* genes, while the probe id for *EPSPS* from other plant species were unavailable. The data obtained in different stress conditions along with their response in different anatomical and developmental stages of plant was retrieved as heat maps (Figure 5A, B, C and D). Relative fold induction of more than 2-folds and decrease of less than 0.5 fold in relative expression was taken as standard cut-off for upregulation and down regulation of the genes, respectively. Most of the stress conditions had only marginal effect on the expression of *EPSPS* genes except for the heat stress on Arabidopsis *EPSPS* gene (AT1G48860) and biotic stress on rice *EPSPS* (LOC_Os06g04280). Pathogens and some elicitors have been found to affect the expression of plant genes for proteins in the shikimate pathway [22-25]. Expression of DHS2, which encodes 3-deoxy-D-arabino heptulosonate (DAHP) synthase, a member of the shikimate pathway, is upregulated by wounding or pathogen attack in Arabidopsis [22]. Moreover, expression of genes encoding DAHP synthase, shikimate kinase (SK., EC 2.7.1.71), 5-enolpyruvyl shikimate 3-phosphate synthase and phenylalanine ammonia-lyase were found to be induced in cultured tobacco cells by elicitor treatment [23]. Apart from these, slight perturbation in rice and Arabidopsis *EPSPS* expression levels were observed under drought, salt and cold stresses. Among hormones, only gibberellic acid treatment altered the expression of two rice *EPSPS* genes (LOC_Os04g31910 and LOC_Os06g04080). The expression analysis of *EPSPS* genes in different plant anatomical features showed higher expression of the gene in root tissues as compared to the aerial part. Overall LOC_Os60g04080 showed very low expression in organ specific and developmental stage specific analysis, whereas LOC_Os04g31910 and LOC_Os06g04280 exhibited moderate expression throughout life cycle with LOC_Os04g31910 showing up-regulation in the senescence stage. In case of Arabidopsis, the *EPSPS* gene expression was highly up-regulated at the initial growth phases (from germination to two-leaf stage) followed by moderate expression during the rest of life cycle. No expression was observed in the siliques. High expression in the initial growth stages in Arabidopsis probably reflects higher requirement of aromatic amino acids at these stages of lifecycle. Overall, the analysis indicates that the gene might play some pivotal roles in maintaining well-being of the plant in different stages of life-cycle as well as under stress conditions. Since the expression profile available from the publically available databases did not account for the effect of glyphosate on the expression profile of *EPSPS* genes, we carried out Real-Time PCR analysis of the *EPSPS* genes in tobacco plant at different time points post Roundup glyphosate (Monsanto) treatment. Upon Real-Time PCR analysis, we observed a significant difference between the expression level of *EPSPS* gene in both time and tissue dependent manner. Interestingly, a significant induced expression of the *EPSPS* gene was observed 6 DPS (days post treatment) which further increased 14 DPS, after which it reduced below the control *EPSPS* expression level (Figure 6A). This reduction was accompanied by senescence phenotype observed in plants after 14 days, while the initial lag phase in expression (till 3DPS) could be attributed to presence of aromatic amino acids in the cellular pool which probably started depleting between 3–6 DPS and hence the *EPSPS* gene was induced to replenish the cellular stocks. In contrast to Arabidopsis and rice *EPSPS* gene expression in young tissues, the qRT-PCR analysis in tobacco revealed significantly induced expression in mature leaves followed by young leaves, shoots and roots, respectively (Figure 6B).

## Conclusions

From the present study, we can conclude that the *EPSPS* family is mainly characterized by the presence of EPSPS I domain which is highly conserved among different plant species. Further, the phylogenetic analysis revealed that the *EPSPS* gene family has diversified in species-specific manner after the monocot-dicot split. The expression analysis showed the differential tissue specific and time dependent expression of *EPSPS* genes which suggested their role in regulating plant growth and its development throughout the life-cycle of plant. Moreover, *in-silico* expression analysis also showed its role in response to various external factors like biotic and abiotic stress. The results presented here is the first detailed study to understand the role of EPSPS in plants. So far, *E. coli EPSPS* gene is the most extensively studied member of the *EPSPS* gene family but its application to develop herbicide tolerant plant has raised a number of ethical and GMO related issues. Therefore, exploration of *EPSPS* genes from plant origin that could aid in crop improvement is the need of the hour.

## Methods

### Identification of EPSPS encoding genes

A preliminary search for *EPSPS* genes was performed using nucleotide sequence of BT022026 (an *EPSPS* from *Arabidopsis thaliana*) as query search for BLAST search (blast.ncbi.nlm.nih.gov) and an e- value of 10^-73^ was chosen as the cut-off for the search. The obtained genes were pooled and redundancy was removed with decrease redundancy software (http://web.expasy.org/decrease_redundancy/). The translated sequences of the candidate genes were further analysed for the presence of specific EPSPS domains and motifs through motifscan (myhits.isb-sib.ch/cgi-bin/motif scan) and scan prosite (Prosite.expasy.nlm.nih.gov) and the genes with characteristic EPSPS domains were shortlisted for further analysis.

### Sequence alignment and phylogenetic analysis

Multiple sequence alignment of the amino acid sequences was obtained using Clustal W (http://www.ebi.ac.uk/Tools/msa/clustalw2/) (Additional file 1). After manually removing the partial sequences, sequence alignment for phylogenetic tree construction was carried out using NCBI COBALT (http://www.ncbi.nlm.nih.gov/tools/cobalt/cobalt.cgi) [26] with default parameters. The fasta file thus generated was analyzed, bootstrapped with 1000 replicates and edited in Mega version 5 programme [27].

### Analysis of EPSPS localization and structure

All predicted EPSPS amino acid sequences were analysed for their sub-cellular localizations via Target p 1.1 (http://www.cbs.dtu.dk/services/TargetP/) [28]. The conserved motifs were identified using MEME suite- GLAM 2, version 4.8.0 using default parameters (http://meme.nbcr.net/meme/) [29]. Homology modelling of EPSPS protein was conducting by using ESyPred web server 1.0 (http://www/fundp.ac.be/sciences/biologie/urbm/bioinfo/esypred/). Molecular graphics visualization programme (PyMol, http://www.pymol.org,) was used for visualization and editing of the generated PDB model.

### Interacting partners and its co-expressed genes

Interacting partners of EPSPS and its co-expressed genes were predicted with String software (http://string-db.org/) [30].

### Gene expression analysis by microarray

*In-silico* expression profile of the *EPSPS* gene was analyzed at developmental and anatomical level and under various stress conditions by retrieving the expression values from affymetrix array database from Genevestigator response viewer (https://www.genevestigator.com/gv/) [31]. ATH1-22 K and Os-51 K: Rice genome 51 K arrays were chosen for the analysis of microarray data for Arabidopsis and rice respectively. For both the plants, microarray data from only the wild type background was analyzed.

### Plant growth parameters and quantitative PCR analysis

Tobacco seedlings were grown in vermiculite in controlled environmental conditions of 200 μmol m^-2^ s^-1^ illumination with day and night cycle of 14 h (25°C)/8 h (18°C). After fourteen days of seed germination, 5–6 leaf staged tobacco plants were sprayed with 2.16 mg/l glyphosate (Roundup® 607 g/l (50.9 w/w), Monsanto Company, St. Louis, MO) [31] for different time points viz., 1, 3, 6, 14, 20 and 48 days. However, 14 days glyphosate treated seedlings were further divided in to root, shoot, young and mature leaves to carry out expression analysis, depending upon the experimental requirement, frozen immediately in liquid nitrogen and stored at −80°C for RNA isolation. Total RNA was isolated from the tobacco plants by TRIzol reagent (Sigma Aldrich, USA) according to the manufactures instructions. Quantitative PCR analysis was performed using *EPSPS* gene specific primers (Forward 5′-TTGCCATGACTCTTGCCGTTGTTG-3′ and Reverse 5′- AAGGCCCGGACTACTGCATTATCA-3′) as described in Garg et al.; [32]. Three biological replicates were chosen for each sample for the expression analysis (n = 9). The expression of *EPSPS* gene in different samples was normalized with the expression of actin (Forward 5′- TGGTCGTACCACCGGTATTGTGTT-3′ and Reverse 5′- CCACGCTCG GTAAGGATCTTC ATC -3′) as the reference gene. The relative mRNA expression was calculated using delta C_T_ method as described by Livak and Schmittgen (2001) [33].

The experimental research on plants have been performed with the approval of an appropriate ethics committee: Review Committee on Genetic Manipulation (RCGM)of Department of Biotechnology, Government of India (Ref. No.: BT/17/06/96-PID; dated 14/6/2012).

## Abbreviations

EPSPS: 5-Enolpyruvylshikimate-3-phosphate synthase; qRT-PCR: quantitative Real-Time Polymerase Chain Reaction; PEP: Phosphoenolpyruvate; Arabidopsis: *Arabidopsis thaliana*; GLP: N-Posphonomethyl- glycine; E.coli: *Escherichia coli*; 3-D: Three dimentional; pI: Isoelectric point; DHS2 Synthase: 3-Deoxy-D-arabino heptulosonate synthase; NJ: Neighbour joining; DPS: Days post spray.

## Competing interests

Authors declare that they have no competing interests.

## Authors’ contributions

BG planned and performed all the experimental work. NV helped in preparation of the final draft of the manuscript. NT supervised the work and helped in the preparation of the final draft of the manuscript. All the authors read and approved the final manuscript.

## Supplementary Material

Additional file 1 Amino acid alignment of EPSPS encoding genes from various organisms by using ClustalW programme. Click here for file
