# Supplementary material for: In-silico analysis and expression profiling implicate diverse role of EPSPS family genes in regulating developmental and metabolic processes
Source: BMC Res Notes. 2014 Jan 22;7:58. doi: 10.1186/1756-0500-7-58 (PMC3903108; doi:10.1186/1756-0500-7-58)
Supplement: Additional file 1 — Amino acid alignment of EPSPS encoding genes from various organisms by using ClustalW programme. [file 1756-0500-7-58-S1.pptx]

## Slide 1
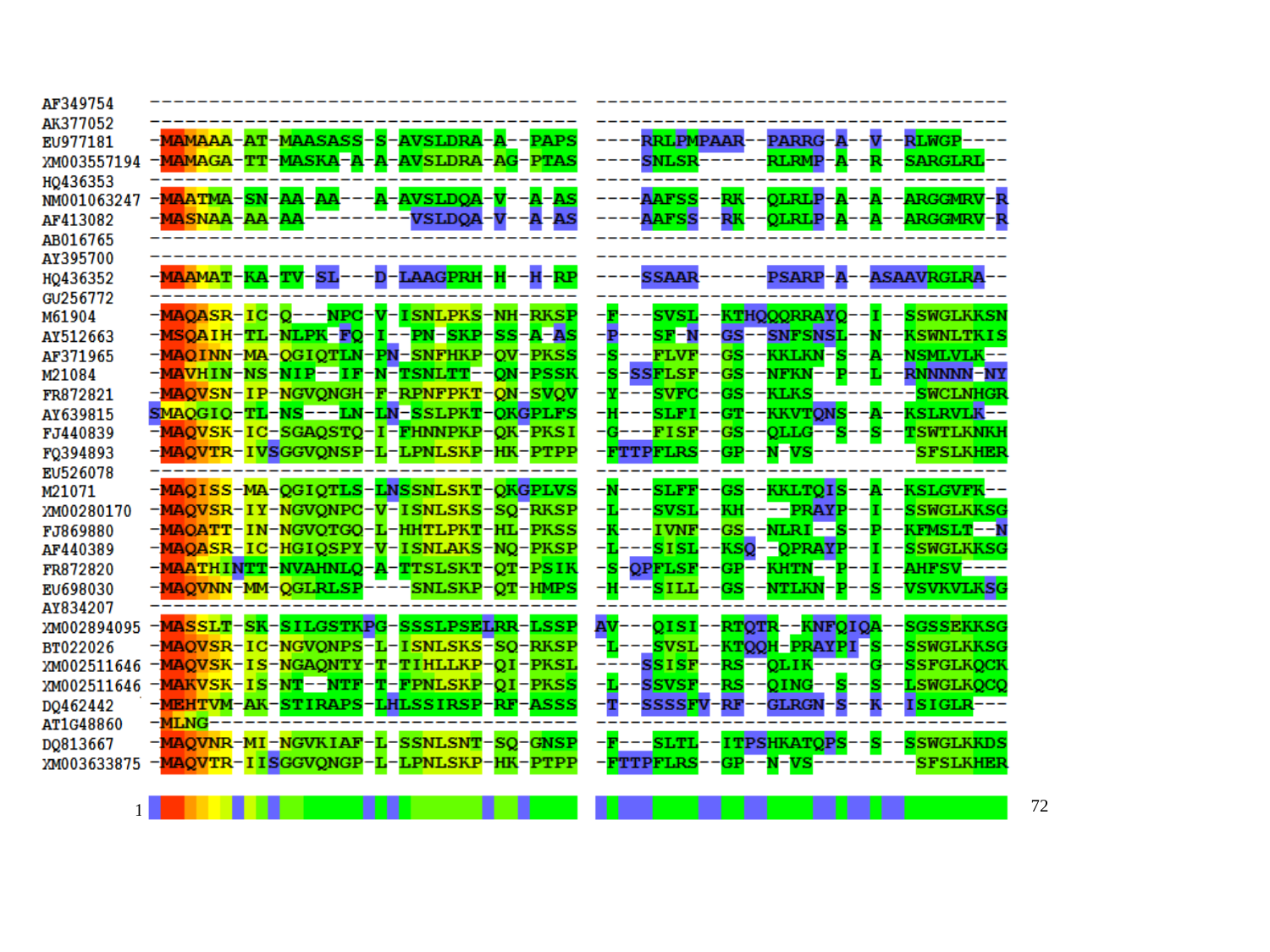

72
1

## Slide 2
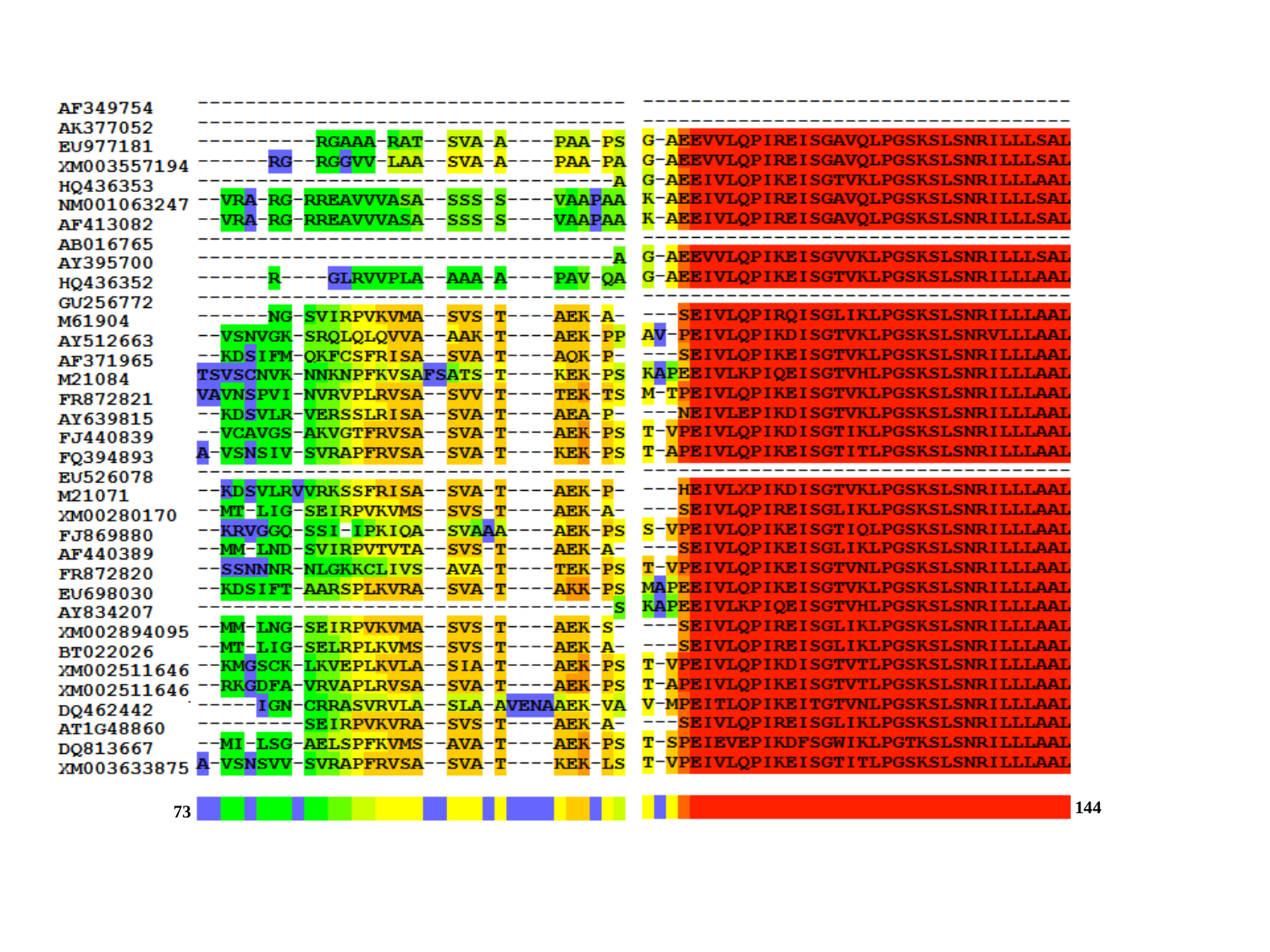

144
73

## Slide 3
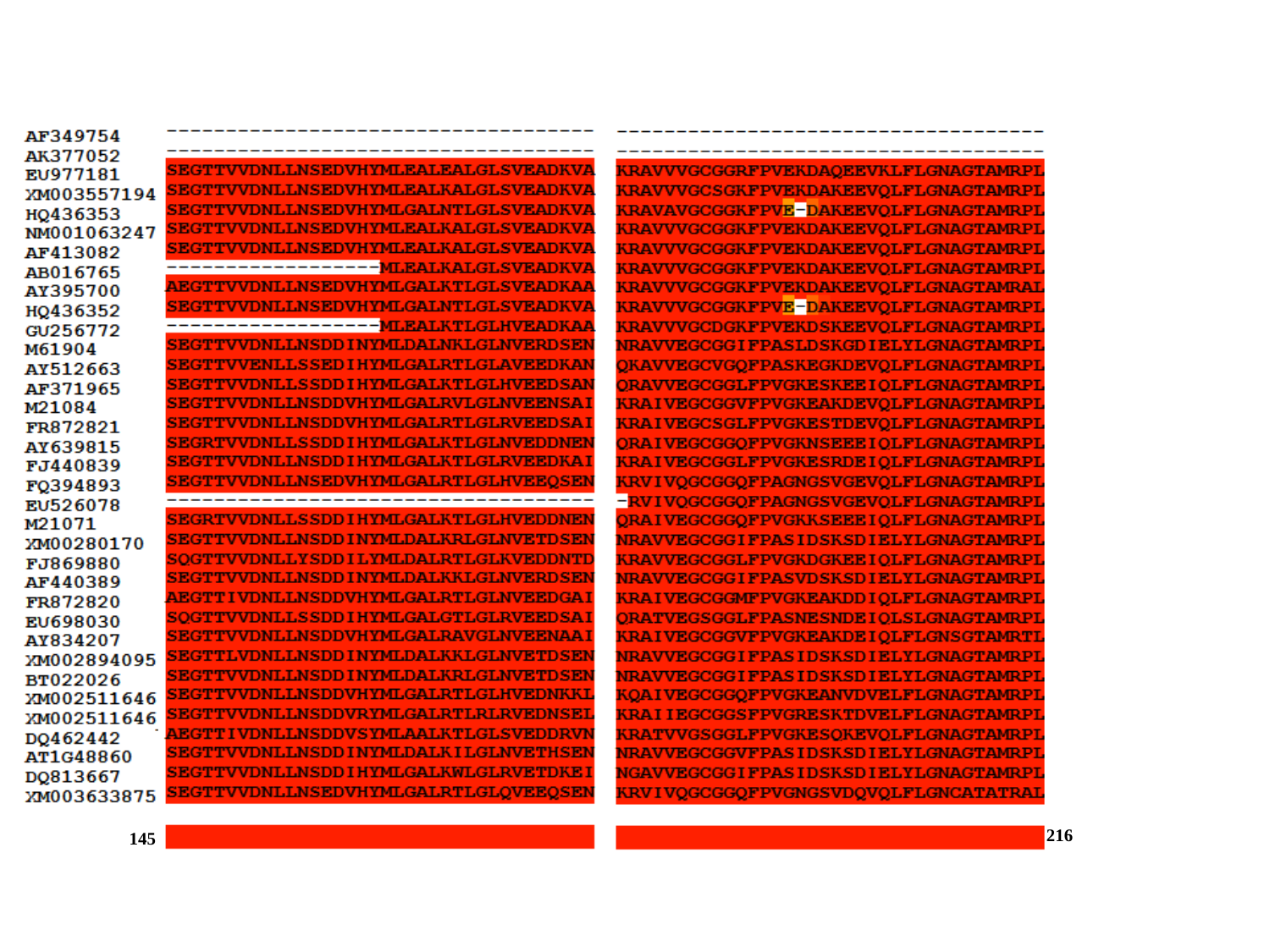

216
145

## Slide 4
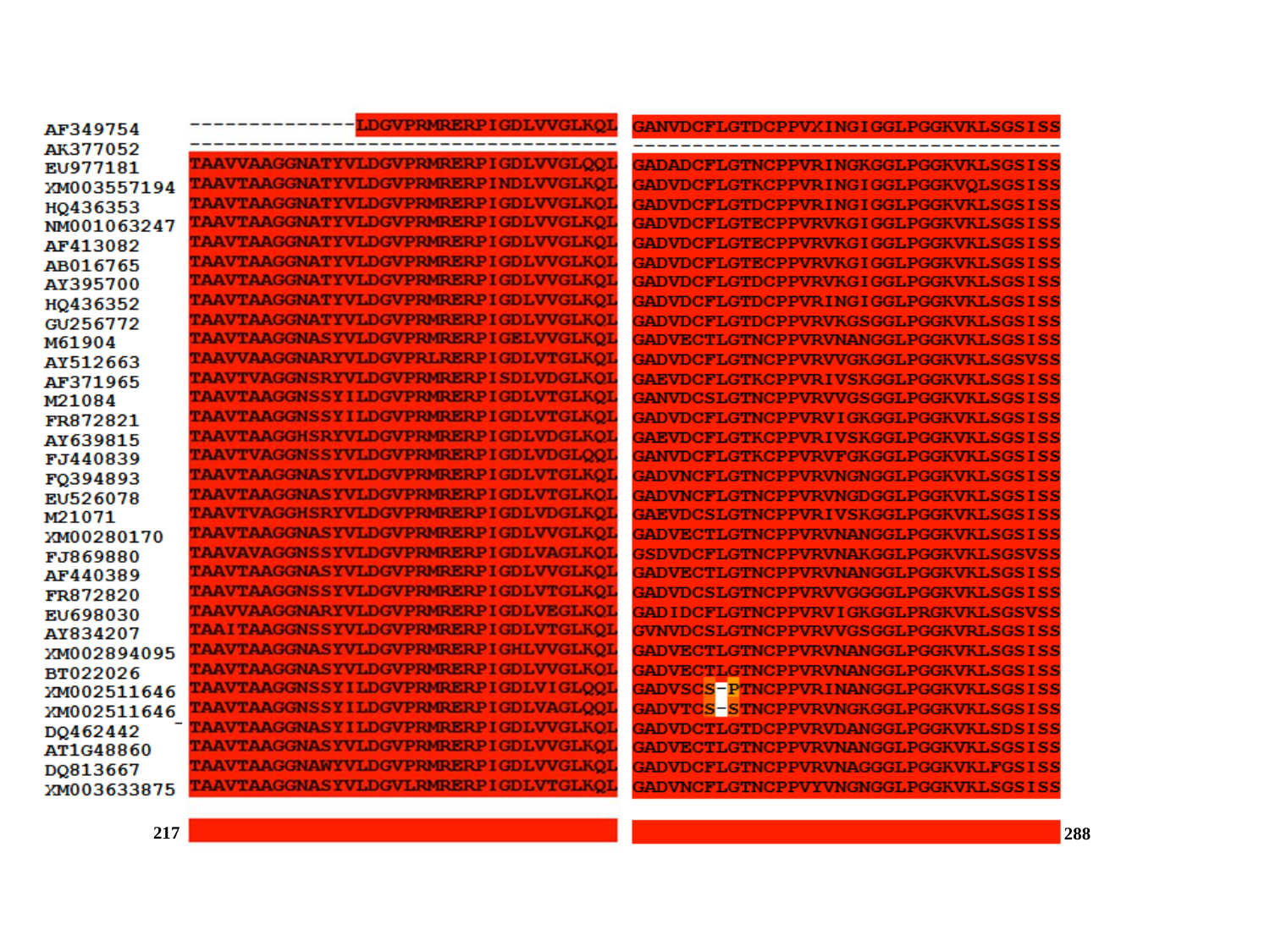

217
288

## Slide 5
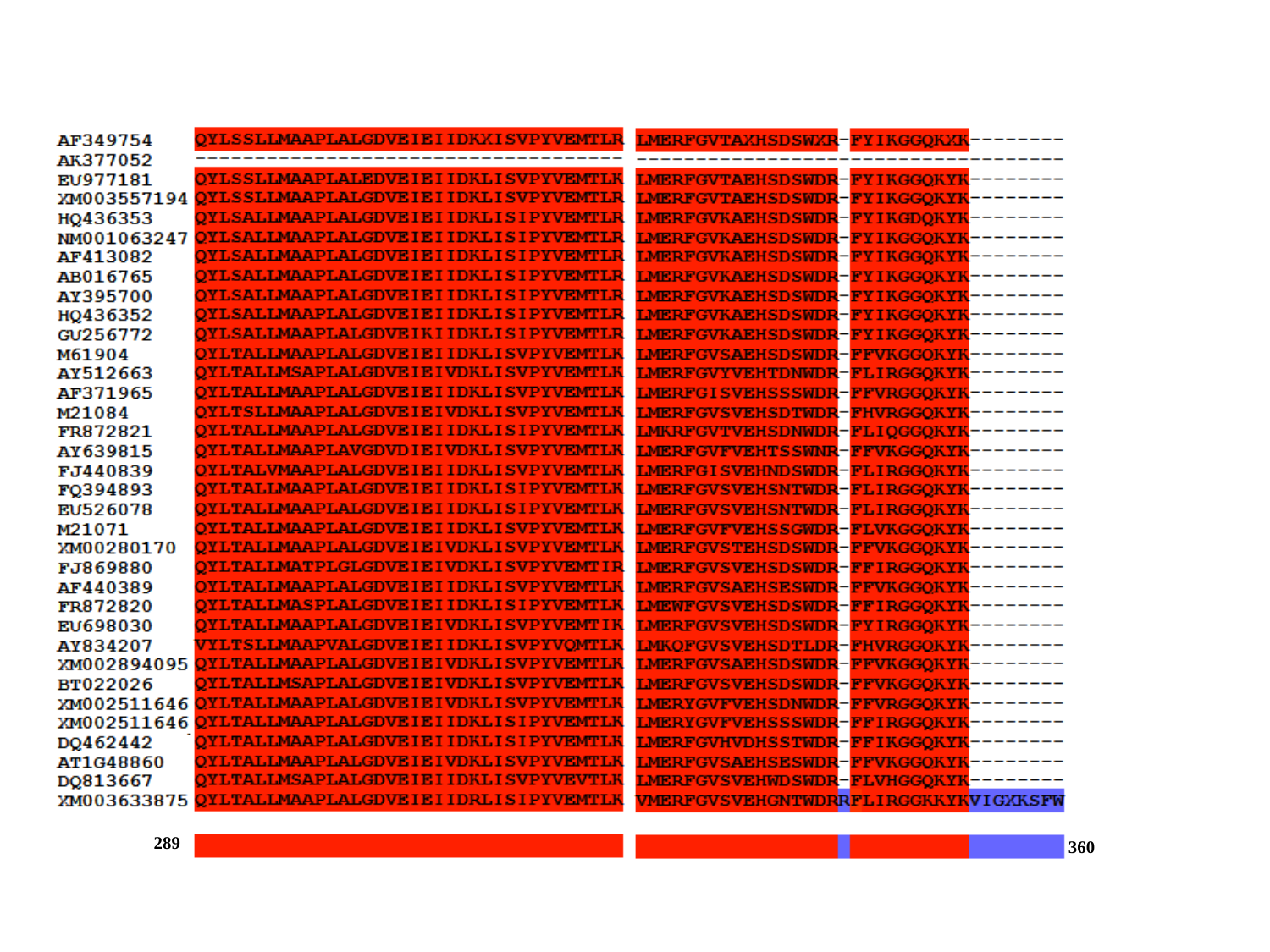

289
360

## Slide 6
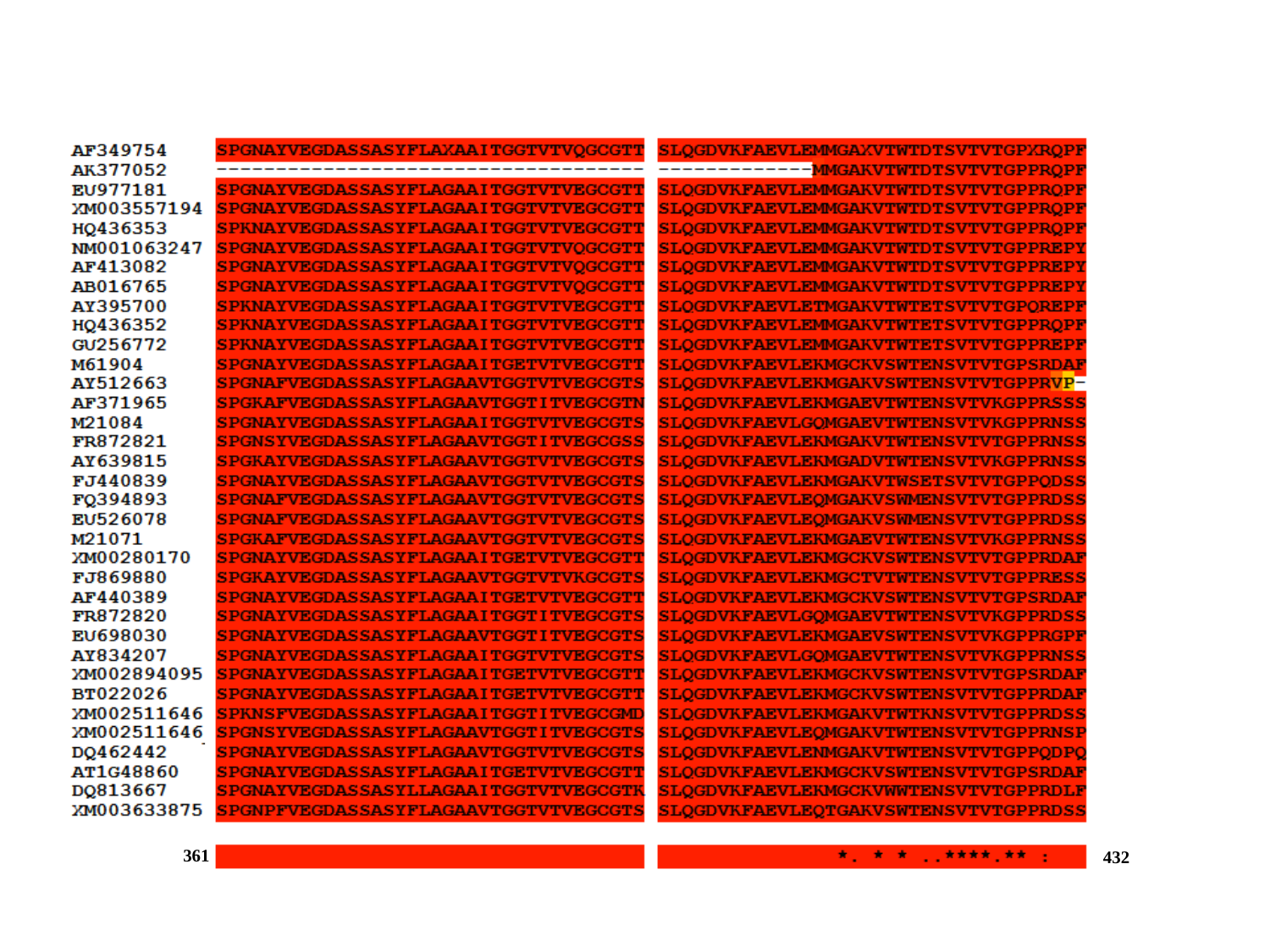

361
432

## Slide 7
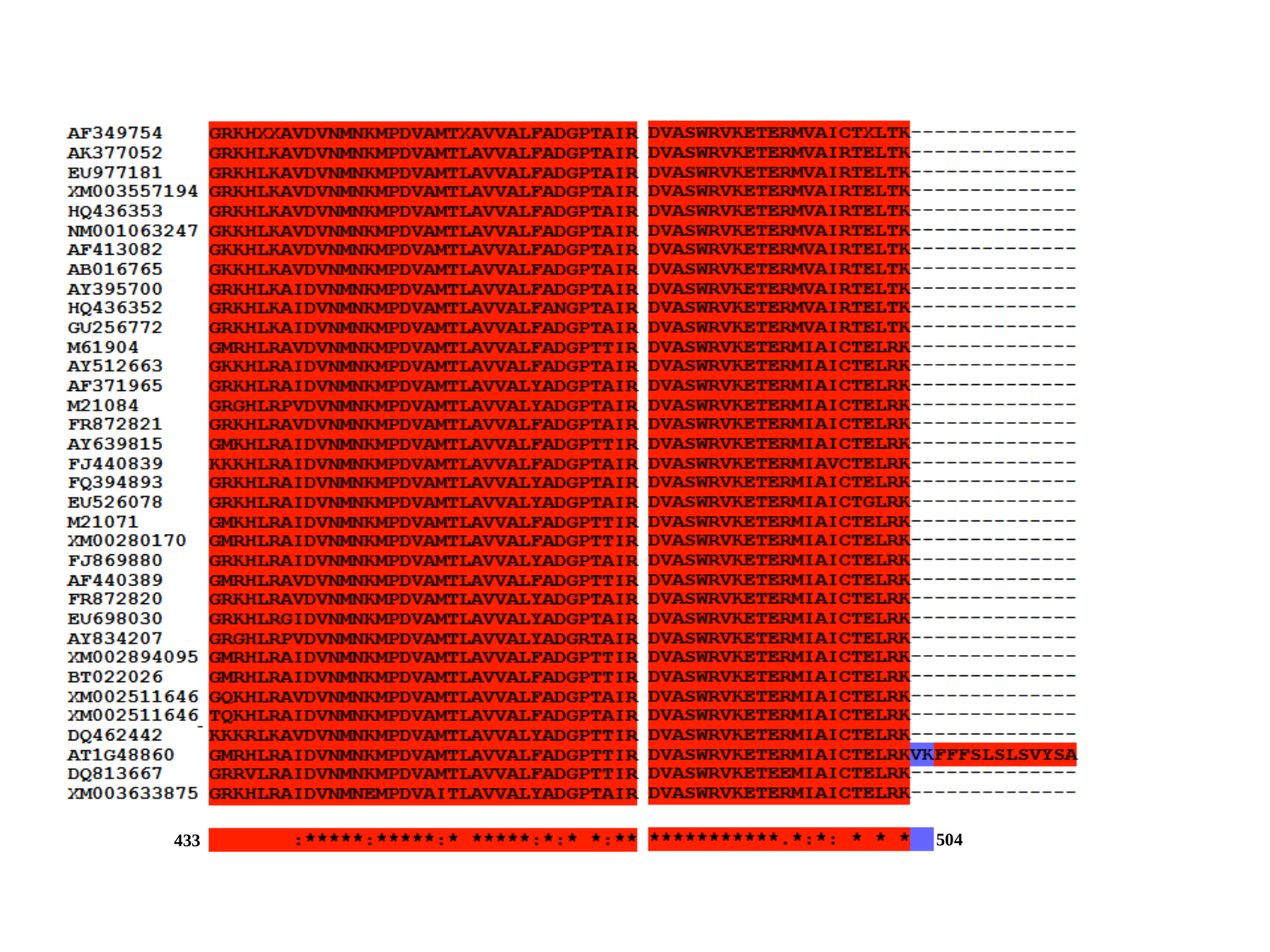

504
433

## Slide 8
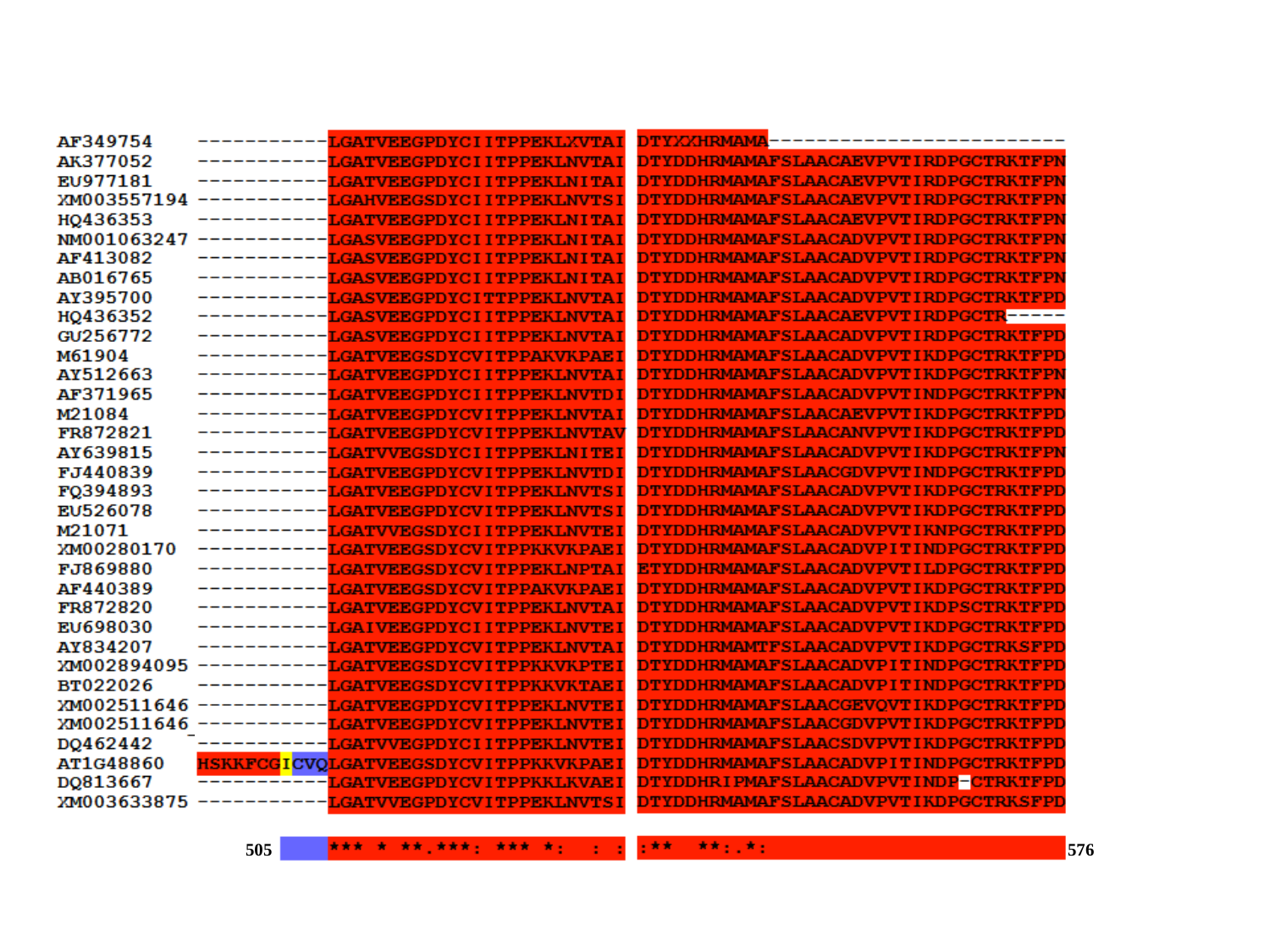

505
576

## Slide 9
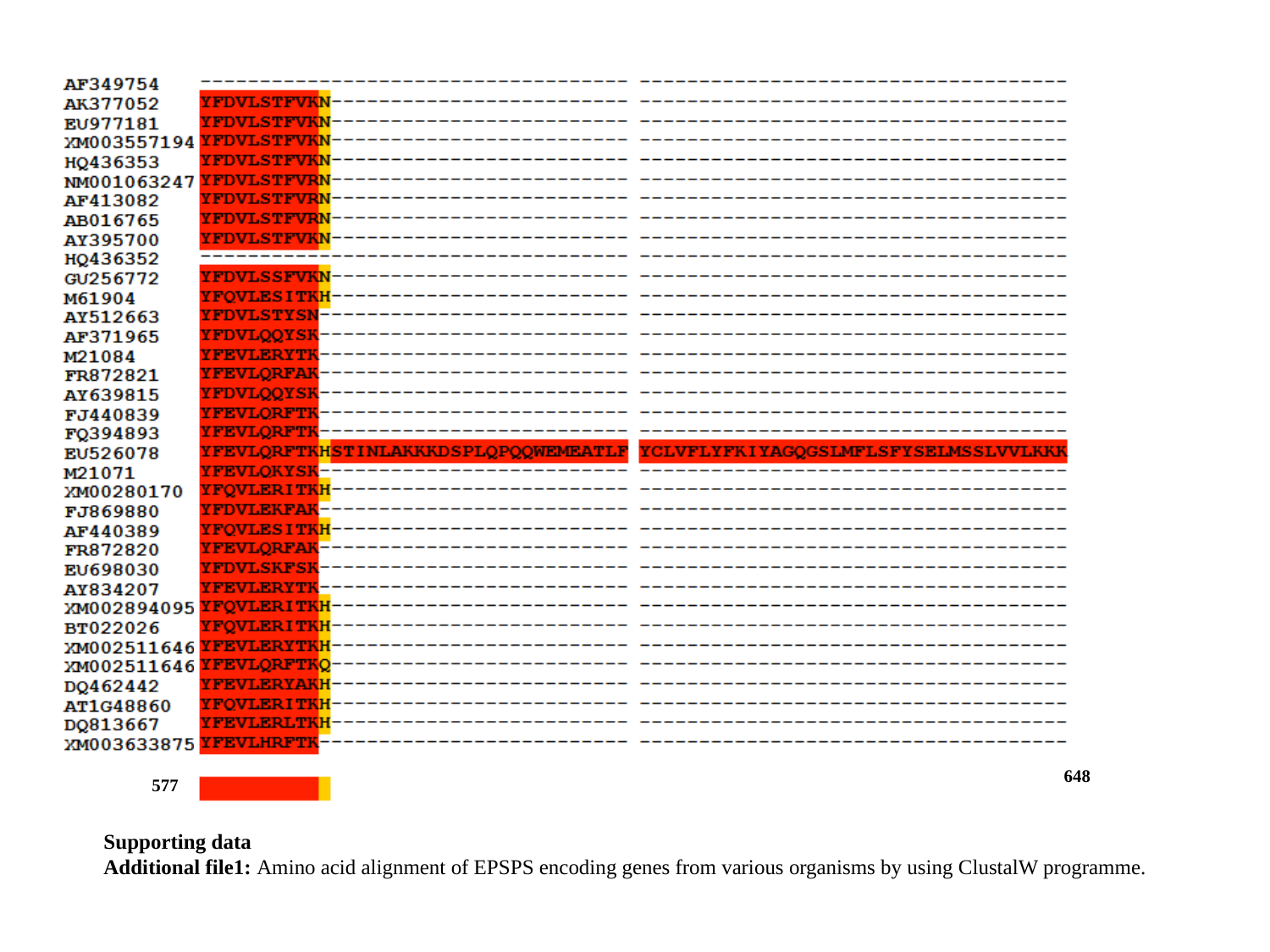

648
577
Supporting data
Additional file1: Amino acid alignment of EPSPS encoding genes from various organisms by using ClustalW programme.
